# The Risk of Schizophrenia and Child Psychiatric Disorders in Offspring of Mothers with Lung Cancer and Other Types of Cancer: A Danish Nationwide Register Study

**DOI:** 10.1371/journal.pone.0079031

**Published:** 2013-11-01

**Authors:** Michael Eriksen Benros, Thomas Munk Laursen, Susanne Oksbjerg Dalton, Merete Nordentoft, Preben Bo Mortensen

**Affiliations:** 1 National Centre for Register-Based Research, Aarhus University, Aarhus, Denmark; 2 Mental Health Centre Copenhagen, Faculty of Health Sciences, University of Copenhagen, Copenhagen, Denmark; 3 The Lundbeck Foundation Initiative for Integrative Psychiatric Research, *i*PSYCH, Aarhus, Denmark; 4 Institute of Cancer Epidemiology, Danish Cancer Society, Copenhagen, Denmark; Baylor College of Medicine, United States of America

## Abstract

**Background:**

Maternal immune responses and brain-reactive antibodies have been proposed as possible causal mechanisms for schizophrenia and some child psychiatric disorders. According to this hypothesis maternal antibodies may cross the placenta and interact with the developing CNS of the fetus causing future neurodevelopmental disorders. Therefore, we investigated if children of mothers with cancer might be at higher risk of developing psychiatric disorders, with particular focus on small-cell lung cancer, which is known to induce production of antibodies binding to CNS elements.

**Methods:**

Nationwide population-based registers were linked, including the Danish Psychiatric Central Register and The Danish Cancer Registry. Data were analyzed as a cohort study using survival analysis techniques. Incidence rate ratios (IRRs) and accompanying 95% confidence intervals (CIs) were used as measures of relative risk.

**Results:**

In general, parental cancer was not associated with schizophrenia in the offspring (IRR, 0.98; 95% CI, 0.95-1.01). Furthermore, we found no temporal associations with maternal cancer in general; neither around the pregnancy period. However, maternal small-cell lung cancer increased the risk of early-onset schizophrenia and maternal small-cell lung cancer diagnosed within 20 years after childbirth increased the risk of schizophrenia. Parental cancer was not associated with child psychiatric disorders (IRR, 1.01; 95% CI, 0.98-1.05) except for the smoking related cancers. There was a significantly increased risk of child psychiatric disorders in offspring of both mothers (IRR, 1.35; 95% CI, 1.16-1.58) and fathers (IRR, 1.47; 95% CI, 1.30-1.66) with lung cancer of all types.

**Conclusions:**

In general, parental cancer did not increase the risk of schizophrenia nor of child psychiatric disorders. However, maternal small-cell lung cancer increased the risk of schizophrenia in subgroups; and lung cancer in general increased the risk of child psychiatric disorders, which could be due to risk factors associated with parental smoking.

## Introduction

Psychiatric disorders have complex, multifactorial, and to a large extent unknown etiologies, but immunological hypotheses have become increasingly prominent. It has been suggested that subgroups of schizophrenia and some child psychiatric disorders, such as autism and Obsessive Compulsive Disorder (OCD), are of a neurodevelopmental origin, where fetal infections and inflammatory mechanisms play an important role [1–5]. Since the increased risk has been associated with several infectious agents, it is hypothesized that it is in fact the maternal immune response that may affect the fetus [6]. Maternal antibodies can cross the placenta and potentially react against the developing nervous system of the fetus, and might induce psychiatric disorders that become clinically manifest many years later [6–11]. Furthermore, elevated levels of antibodies have been found at the end of pregnancy in mothers of children who later develop schizophrenia [5], and brain-reactive antibodies have been found in pregnant mothers and their children who later develop autism [12–14].

Several autoantibodies produced by the immune system as a response to cancer, infections or autoimmune diseases can react against brain tissue, and are suggested to be implicated in inducing psychiatric symptoms [15–17]. Brain-reactive antibodies are particularly known to be involved in the etiology of paraneoplastic CNS-symptoms, which may in part be caused by an immune response where antibodies against tumor antigens cross-react with elements of the nervous system [18,19]. Particularly small-cell lung cancer can produce psychiatric pathology in the affected individual [20,21]; and immunologically, small-cell lung cancer has a strong resemblance to brain tissue [22,23]. All small-cell lung cancers seem to express neuronal proteins such as Hu [24,25]; however, for instance, anti-Hu reactivity has only been found in 17-25.5% of cases with small-cell lung cancer [26,27]. Nonetheless, several other autoantibodies with brain-reactive potential are associated with small-cell lung cancer [18]; and some autoantibodies in mothers with small-cell lung cancer might also be able to react with the nervous system of the fetus, predisposing for future psychiatric disorders in the offspring [6,7,15]. In a previous population based Danish study by Dalton et al. [28] looking at a possible familial association, positive or negative, between schizophrenia and cancer, the incidence of lung cancer was significantly increased in mothers to schizophrenic offspring but not in fathers to schizophrenic offspring, when compared to parents in general. In other population based studies by Lichtermann et al. [29], Levav et al. [30] and Ji et al.[31], no significantly increased risks were observed when using the general population as a comparison group instead of parents; and therefore not considering a possible “healthy parenthood effect” [32–34]. None of the previous studies evaluated a possible difference between the types of lung cancer or a possible temporal association. 

The aim of this study was to investigate the associations between maternal cancer and psychiatric disorders based on the nationwide Danish registers, in order to indirectly study the possible involvement of maternal autoantibodies and immune responses in the development of psychiatric disorders. In cancer patients, autoantibodies are most common in patients with small-cell lung cancer, so the hypothesis would predict that especially small-cell lung cancer in the mother increases the risk of schizophrenia and some child psychiatric disorders in the offspring. If the risk is elevated for children of fathers with small-cell lung cancer as well, it would probably be attributable to genetic or environmental factors such as smoking. 

## Materials and Methods

### Data sources

The study was based upon linkage between the Danish Civil Registration System (CRS) [35], the Danish Psychiatric Central Register [36,37], and The Danish Cancer Registry[38]. From 1969, the diagnostic system used in the Psychiatric Central Register was the Danish modification of the *International Classification of Diseases*, 8th Revision (ICD-8) [39], and from 1994, the diagnostic system used was the Danish modification of the *International Classification of Diseases*, 10th Revision (ICD-10) [40]. Details of individual cases of cancer are available according to the Danish version of the ICD-7 through 2003 and ICD-10 since 2004. Morphology and topography have been classified by the International Classification of Diseases for Oncology (ICD-O) since 1978 [38]. The described nationwide Danish registers are available to all researchers in Denmark after approval from the Danish Data Protection Agency.

### Study population

All individuals, born in Denmark, who were alive on January 1, 1978 or born between that date and April 1, 2011, were identified. From these, we extracted persons who were registered as parents and having children born after 1955. Due to the differences in age at onset for schizophrenia and child psychiatric disorders, respectively, 2,747,179 persons were included in the study of schizophrenia, compared to 3,162,109 persons included when looking at the risk for child psychiatric disorders, since only persons younger than 15 years were included. The parents’ cancer diagnosis was included in the analyses as exposure and the outcome was a psychiatric disorder in the offspring defined as described below.

All parents were followed using information from the Cancer Registry and CRS, beginning on January 1, 1978, where histological type of the cancer could be identified and censored at the date of death, emigration, or December 31, 2009, whichever came first. In parents, cancer sites of *a priori* interest were lung cancer (ICD-7: 162; ICD-10: C33-C34), which was subdivided into small-cell lung cancer and non-small-cell lung cancer. Small-cell lung cancer was identified using the ICD-7 162 code and ICD-10 C33-C34 code combined with morphology codes 80413, 80419, 80433 or 80733. The other cancer diagnoses were divided into groups of smoking related cancers except for lung cancer (buccal cavity (ICD-7: 143-148; ICD-10: C03-C06, C09-C14), esophagus (ICD-7: 150; ICD-10: C15), pancreas (ICD-7: 157; ICD-10: C25), larynx (ICD-7: 161; ICD-10: C32), kidney (ICD-7: 180; ICD-10: C64) and bladder (ICD-7: 181; ICD-10: C67) cancer) and a group with the remaining cancers. 

Members of the parent cohort and their offspring were linked to the files of the Danish Psychiatric Central Register. Follow-up of offspring began January 1, 1978 and ended April 1, 2011, only including children born after 1955. In the offspring, all psychiatric diagnoses were categorized on the basis of the clinical ICD-8 and ICD-10 main diagnosis. Persons with any psychiatric contact with schizophrenia were identified under ICD-8 code 295 and ICD-10 codes F20. Childhood psychiatric diagnoses were defined as any psychiatric diagnosis in persons aged less than 15 years (ICD-8: 290-315; ICD-10: F00-F99), to ensure comparability between the two diagnostic systems used during the study period. 

### Statistical analysis

Data were analyzed as a cohort study, using survival analysis techniques. The relative risks of schizophrenia and child psychiatric disorders were estimated by a log linear Poisson regression model, which was used as an approximation to the Cox-regression. The main exposure variables were maternal cancer and paternal cancer. We used incidence rate ratios (IRRs) as the measure of relative risk. Tests of significance and 95% confidence intervals (CIs) for the IRRs were based on log-likelihood tests [41]. Separate analyses were made for mothers and fathers, and adjustment was made in all analyses for sex of the offspring, age and calendar year. Analyses were also conducted with further adjustment for in- or outpatient psychiatric contacts by the parent. Power calculations were performed according to the method by Hsieh and Lavori [42], which is implemented in the Stata procedure stpower cox.

## Results

### The risk of schizophrenia

As displayed in Table 1 and 2, a total of 6,029 cases of schizophrenia in children of parents with cancer of all types, were diagnosed from 1978 through 2011, during 55,565,503 person-years of risk. The overall risk of schizophrenia in children of parents with cancer was close to unity (IRR, 0.98; 95% CI, 0.95-1.01), compared to children of parents with no cancer diagnosis during our study period. A total of 929 cases of schizophrenia had a parent with lung cancer, corresponding to an IRR of 1.06 (95% CI, 0.96-1.18) for children of mothers with lung cancer, and an IRR of 1.06 (95% CI, 0.97-1.15) for children of fathers with lung cancer. Looking at the specific IRR of schizophrenia in children of parents with small-cell lung cancer, we found that the IRR was non-significantly elevated to 1.15 (95% CI, 0.91-1.46) among children of mothers with small-cell lung cancer based on 71 cases, but not among children of fathers with small-cell lung cancer (IRR, 0.93; 95% CI, 0.75-1.15) based on 84 cases (Table 2). However, in subgroup analyses, the risk of schizophrenia in offspring to mother’s with small-cell lung cancer was significantly elevated in the group with early onset schizophrenia before the age of 30 (IRR, 1.35; 95% CI, 1.01-1.80) based on 47 cases, particularly in the age group younger than 25 years (IRR, 1.70; 95% CI, 1.22-2.39) based on 34 cases and also after adjusting for a psychiatric family history (IRR, 1.54; 95% CI, 1.10-2.16). When looking at children of parents with all other cancer types than lung cancer, the overall risk of schizophrenia was close to unity in children of mothers (IRR, 0.99; 95% CI, 0.95-1.03) and slightly, but significantly decreased in children of fathers with all other cancer types than lung cancer (IRR, 0.94; 95% CI, 0.90-0.98), compared to children of parents with no cancer diagnosis during our study period.

**Table 1:** Characteristics of the population-based cohorts with schizophrenia and child psychiatric disorders in Denmark, 1978-2011.

**Characteristics of 19505 cases with schizophrenia in a population-based cohort of 2.75 million Danish people**		
**Variable**	**No.**	**%**
Total cases	19505	100.00
Sex		
Male	1407824	51.25
Female	1339355	48.75
Mean age at entry (years)	15.84	
Mean age at entry cases (years)	16.04	
Mean follow-up (years)	20.23	
History		
Maternal cancer	3045	15.61
Paternal cancer	2984	15.30
Parental psychiatric contact	7167	36.74
Urbanicity at birth		
Capital	433734	15.79
Capital suburb	285935	10.41
Provincial city	346289	12.61
Provincial town	913713	33.26
Rural areas	767508	27.94
**Characteristics of 61950 cases with child psychiatric disorders in a population-based cohort of 3.16 million Danish people**		
**Variable**	**No.**	**%**
Total cases	61950	100.00
Sex		
Male	1622119	51.30
Female	1539990	48.70
Mean age at entry (years)	2.73	
Mean age at entry cases (years)	0.26	
Mean follow-up (years)	9.82	
History		
Maternal cancer	2231	3.60
Paternal cancer	2011	3.25
Parental psychiatric contact	21437	34.60
Urbanicity at birth		
Capital	481379	15.22
Capital suburb	373738	11.82
Provincial city	407512	12.89
Provincial town	1004390	31.76
Rural areas	895090	28.31

**Table 2:** Incidence rate ratios (IRR) of schizophrenia in offspring to parents with cancer, adjusted for sex, age, and calendar period, Denmark, 1978-2011.

**Schizophrenia**	Schizophrenia risk, Relative to risk in Offspring to Parents without cancer		Also adjusted for parents previous psychiatric contacts		
**Type of cancer in the mother**	**IRR**	**CI**	**IRR**	**CI**	**Cases**
Lung cancer, all types	1.06	0.96-1.18	1.01	0.91-1.13	360
Small-cell lung cancer (SCLC)	1.15	0.91-1.46	1.09	0.87-1.38	71
Non-SCLC	1.04	0.93-1.17	1.00	0.89-1.12	289
All cancers except lung	0.99	0.95-1.03	1.01	0.96-1.05	2,685
Smoking related except lung cancer^1^	1.06	0.94-1.21	1.04	0.91-1.18	245
All cancers except smoking related	0.98	0.94-1.03	1.00	0.96-1.05	2,440
No cancer (ref)	1.00	-	1.00	-	16,460

**Type of cancer in the father**	**IRR**	**CI**	**IRR**	**CI**	**Cases**
Lung cancer, all types	1.06	0.97-1.15	1.04	0.95-1.13	569
Small-cell lung cancer	0.93	0.75-1.15	0.90	0.73-1.12	84
Non-SCLC	1.09	0.99-1.19	1.07	0.97-1.17	485
All cancers except lung	**0.94**	0.90-0.98	**0.94**	0.90-0.99	2,415
Smoking related except lung cancer^1^	0.96	0.88-1.04	0.94	0.86-1.02	597
All cancers except smoking related	**0.93**	0.89-0.98	0.95	0.90-1.00	1,818
No cancer (ref)	1.00	-	1.00	-	16,521

^1^ Cancer in the buccal cavity, esophagus, pancreas, larynx, kidney and bladder.

Adjusting for parents’ history of psychiatric disorders made little difference to the IRR estimates in offspring of parents with cancer as shown in Table 2. Additional adjustments for parental age and urbanicity at childbirth did not change the risk estimates (data not shown). During the study period, the overall incidence rate ratio of schizophrenia was 1.67 times higher in males than in females when adjusted for age and calendar year. The study has a power of 80% to detect an effect of maternal cancer on the offspring’s risk of schizophrenia with an IRR of less than 0.94 or greater than 1.06. When looking at maternal small-cell lung cancer the power was 80% to detect an IRR of less than 0.67 or greater than 1.49.

The temporal associations between maternal cancer diagnosis and childbirth did not in general affect the risk of schizophrenia (p=0.233), as demonstrated in Table 3. However, the risk of schizophrenia was elevated if the small-cell lung cancer in the mother was diagnosed within 20 years after giving birth to the offspring, but only based on 8 cases (IRR, 2.76; 95% CI, 1.38-5.52). 

**Table 3:** Incidence rate ratios (IRR) of schizophrenia and child psychiatric disorders according to the temporal associations to a parental cancer diagnosis, adjusted for sex, age, and calendar period, Denmark, 1978-2011.

**The risk of schizophrenia and the temporal associations with a parental cancer diagnosis**							
**The child’s age at the mother’s cancer diagnosis**	**IRR**	**95%CI**	**Cases**	**The child’s age at the father’s cancer diagnosis**	**IRR**	**95%CI**	**Cases**
0-10 years of age	1.10	0.95-1.28	179	0-10 years of age	0.98	0.82-1.17	121
10-20 years of age	1.10	1.00-1.20	489	10-20 years of age	1.04	0.94-1.16	350
20-30 years of age	0.98	0.90-1.08	506	20-30 years of age	0.96	0.87-1.05	463
30+ years of age	0.99	0.89-1.10	364	30+ years of age	0.93	0.83-1.03	389
Persons without a parent with cancer (reference)	1.00		17,936	Persons without a parent with cancer (reference)	1.00		18,143
**The risk of child psychiatric disorders and the temporal associations with a parental cancer diagnosis**							
**The child’s age at the mother’s cancer diagnosis**	**IRR**	**95%CI**	**Cases**	**The child’s age at the father’s cancer diagnosis**	**IRR**	**95%CI**	**Cases**
0-10 years of age	1.04	0.96-1.13	585	0-10 years of age	**1.15**	1.05-1.26	472
10-15 years of age	0.98	0.84-1.15	154	10-15 years of age	**1.28**	1.08-1.52	137
Persons without a parent with cancer (reference)	1.00		61,211	Persons without a parent with cancer (reference)	1.00		61,341

### The risk of child psychiatric disorders

As displayed in Table 1 and 4, a total of 4,242 cases of child psychiatric disorders were diagnosed in offspring of parents with cancer during our study period, resulting in 31,047,212 person-years of risk. The overall risk of child psychiatric disorders in offspring to parents with cancer was close to unity (IRR, 1.01; 95% CI, 0.98-1.05), compared to children of parents with no cancer diagnosis during our study period. A total of 422 cases with child psychiatric disorders had a parent with lung cancer, corresponding to an elevated IRR of child psychiatric disorders with 1.35 (95% CI, 1.16-1.58) if the mother had lung cancer and almost similarly elevated to 1.47 (95% CI, 1.30-1.66) if the father had lung cancer, compared to children of parents with no cancer diagnosis during our study period. After adjustment for psychiatric history of the parents, the risk of child psychiatric disorders remained significantly elevated in offspring to parents with lung cancer as shown in Table 4. Furthermore, adjustments for parental age and urbanicity at childbirth did not change the risk estimates (data not shown). When looking specifically at small-cell lung cancer in the parent, we only found 24 cases of child psychiatric disorders in offspring of mothers diagnosed with small-cell lung cancer during the study period, resulting in an non-significantly elevated IRR of 1.47 (95% CI, 0.98-2.19). There were 42 cases of child psychiatric disorders in offspring of fathers diagnosed with small-cell lung cancer, resulting in a significantly elevated IRR of 1.36 (95% CI, 1.01-1.84). The risk of child psychiatric disorders was significantly elevated in both offspring of mothers with non-small-cell lung cancer (IRR, 1.34; 95% CI, 1.13-1.58) and in offspring of fathers with non-small-cell lung cancer (IRR, 1.49; 95% CI, 1.31-1.70), compared to children of parents with no cancer diagnosis during our study period. When looking at offspring of parents with all cancer types excluding lung cancer, the risk were close to unity in offspring of mothers (IRR, 0.97; 95% CI, 0.93-1.01) and in offspring of fathers (IRR, 1.01; 95% CI, 0.96-1.05). However, the risk of child psychiatric disorders was also elevated in offspring of fathers with other smoking related cancers than lung cancer (IRR, 1.17; 95% CI, 1.05-1.30), whereas in offspring of mothers with smoking related cancers except for lung cancer the risk was not significantly elevated (IRR, 1.14; 95% CI, 0.94-1.38). The study has a power of 80% to detect an effect of maternal cancer on the offspring’s risk of child psychiatric disorders with an IRR of less than 0.96 or greater than 1.04. When looking at maternal small-cell lung cancer we had a power of 80% to detect an IRR of less than 0.73 or greater than 1.37.

**Table 4:** Incidence rate ratios (IRR) of child psychiatric disorders in offspring to parents with cancer, adjusted for sex, age, and calendar period, Denmark, 1978-2011.

**Child psychiatric disorders**	Risk of child psychiatric disorders, Relative to risk in Offspring to parents without cancer		Also adjusted for parents previous psychiatric contacts		
**Type of cancer in the mother**	**IRR**	**CI**	**IRR**	**CI**	**Cases**
Lung cancer, all types	**1.35**	1.16-1.58	**1.25**	1.07-1.46	158
Small-cell lung cancer (SCLC)	1.47	0.98-2.19	1.30	0.87-1.94	24
Non-SCLC	**1.34**	1.13-1.58	**1.24**	1.05-1.47	134
All cancers except lung	0.97	0.93-1.01	0.97	0.93-1.02	2,073
Smoking related except lung cancer^1^	1.14	0.94-1.38	1.06	0.87-1.28	106
All cancers except smoking related	0.96	0.92-1.00	0.97	0.92-1.01	1,967
No cancer (ref)	1.00	-	1.00	-	59,719

**Type of cancer in the father**	**IRR**	**CI**	**IRR**	**CI**	**Cases**
Lung cancer, all types	**1.47**	1.30-1.66	**1.35**	1.20-1.53	264
Small-cell lung cancer	**1.36**	1.01-1.84	1.23	0.91-1.67	42
Non-SCLC	**1.49**	1.31-1.70	**1.38**	1.21-1.57	222
All cancers except lung	1.01	0.96-1.05	1.00	0.95-1.04	1,747
Smoking related except lung cancer^1^	**1.17**	1.05-1.30	1.10	0.99-1.22	356
All cancers except smoking related	0.97	0.92-1.02	0.97	0.92-1.03	1,391
No cancer (ref)	1.00	-	1.00	-	59,939

^1^ Cancer in the buccal cavity, esophagus, pancreas, larynx, kidney and bladder.

The temporal associations between maternal cancer diagnoses and child birth did not in general affect the risk of child psychiatric disorders (p=0.615) as displayed in Table 3.

To further assess the specific child psychiatric diagnoses in offspring of parents with lung cancer, we restricted the study period to 1994-2011 during which ICD-10 was used to classify psychiatric diagnoses, thus securing comparable diagnoses. As shown in Table 5, the IRR of a child psychiatric diagnosis within the ICD-10 diagnoses F90-F98 (behavioural and emotional disorders with onset usually occurring in childhood and adolescence) was elevated to 1.43 (95% CI, 1.16-1.77) if a parent had lung cancer, compared to children of parents with no cancer diagnosis during our study period. The IRR of child psychiatric disorders within the ICD-10 diagnoses F40-F48 (neurotic, stress-related and somatoform disorders) was elevated with an IRR of 1.74 (95% CI, 1.33-2.26) if a parent had lung cancer.

**Table 5:** Incidence rate ratios (IRR) subdivided by type of ICD-10 child psychiatric disorders in offspring to parents with lung cancer, adjusted for sex, age, and calendar period, Denmark, 1994-2011.

	**Child psychiatric diagnosis in offspring to parents with lung cancer**											
	**F40-48 Neurotic, stress-related and somatoform disorders**			**F80-F89 Disorders of psychological development**			**F90-98 Behavioural and emotional disorders with onset usually occurring in childhood and adolescence**			**Other diagnosis**		
Type of cancer	IRR	95% CI	Cases	IRR	95% CI	Cases	IRR	95% CI	Cases	IRR	95% CI	Cases
**Lung cancer in the mother**	1.55	0.99-2.43	19	1.13	0.64-1.98	12	1.36	0.96-1.93	32	1.25	0.82-1.89	22
No lung cancer in the mother (reference)	1.00	-	8,940	1.00	-	12,426	1.00	-	21,864	1.00	-	13,504
**Lung cancer in the father**	**1.89**	1.37-2.61	37	1.38	0.93-2.07	24	**1.47**	1.13-1.92	55	**1.71**	1.29-2.28	48
No lung cancer in the father (reference)	1.00	-	8,922	1.00	-	12,414	1.00	-	21,841	1.00	-	13,478
**Lung cancer in the parent**	**1.74**	1.33-2.26	55	1.29	0.93-1.79	36	**1.43**	1.16-1.77	87	**1.51**	1.20-1.92	69
No lung cancer in the parent (reference)	1.00	-	8,904	1.00	-	12,402	1.00	-	21,809	1.00	-	13457

## Discussion

We set out to study if the mother’s immune system could affect the offspring, and therefore looked at parents exposed to cancer and their children’s risk of schizophrenia and child psychiatric disorders. The cancer might affect the immune system and the production of autoantibodies many years before detection, since the process of developing cancer has been initiated much earlier. We focused on the subtypes of lung cancer, since particularly small-cell lung cancer is known to induce the production of autoantibodies that can cross-react with brain tissue [18–21,23]. Maternal antibodies can cross the placenta and cause neonatal disease or even alter the development of the infant, indicating that some neurodevelopmental disorders could be caused by maternal antibodies reacting against the developing nervous system of the fetus [6,7,10,11,43,44]. 

### Schizophrenia

Compared to the previous Danish study on the associations between parental cancer and schizophrenia with a follow-up until 1997[28], we had follow-up until 2011, and furthermore focused on the subtypes of lung cancer with emphasis on small-cell lung cancer. We found that in this updated study, there was no longer an overall association between maternal lung cancer and schizophrenia. However, subgroup analyses indicated that maternal small-cell lung cancer increased the risk of early-onset schizophrenia (under the age of 30) and also if the mother was diagnosed with small-cell lung cancer within 20 years after giving birth to the child, but based on few cases. 

No general temporal association was found between maternal cancer and the risk of schizophrenia. The findings that a maternal immune activating event such as cancer did not in general increase the risk of schizophrenia, is in line with a recent Danish epidemiological study of parental infections by Nielsen et al.[45], showing no significant difference in the increased risk of schizophrenia if the infections occurred during or outside the pregnancy period; and the risk of schizophrenia was similarly increased when comparing infections in the mother and father. Furthermore, another Danish register study by Eaton et al.[46] found that parental autoimmune diseases increased the risk of schizophrenia by 10%, but did not investigate possible differences between maternal or paternal autoimmune diseases. However, other studies have indicated associations between maternal immune response as a potential risk factor for future schizophrenia in the offspring, in line with previous findings of associations between maternal infections and schizophrenia [2,4,47–49].

Our study supported the previous finding that parental cancer in general was not associated with schizophrenia in the offspring [28], and hence, do not support the hypothesis of a genetic association between parental cancer and schizophrenia. Conversely, other population based studies have found a slightly decreased overall cancer risk when using the general population as a comparison group instead of only comparing to parents [29–31], thus not considering a possible “healthy parenthood effect” as we did [32–34]. 

### Child psychiatric disorders

When looking at child psychiatric disorders, the increased risk does not seem to be explained by the immune model, since the risk of child psychiatric disorders was similarly increased if the mother or the father was diagnosed with lung cancer of all types. We found a 35% increased risk of child psychiatric disorders in children of mothers with lung cancer, and a 47% increased risk in children of fathers with lung cancer, with the significance remaining after adjusting for parental mental illness. Common genetic risk factors might be involved in some cancers and child psychiatric disorders, although there is no evidence of this in the literature [50]. Furthermore, offspring to parents with cancer in general did not have an elevated risk of child psychiatric disorders. It is a limitation of our register-based study that we were unable to consider possible differences in risk behaviour between parents in the general population and the parents of children developing psychiatric disorders. It is possible that offspring with child psychiatric disorders might be exposed to more passive smoking, for instance, than the general population. Smoking in mothers during pregnancy has been associated with an increased risk of child psychiatric disorders in their offspring, such as ADHD [51,52]. To further explore the possible effect of smoking, we also looked at other smoking related cancers than lung cancer, and found that the risk of child psychiatric disorders was not significantly increased in offspring to mothers with other smoking related cancers; but in offspring to fathers, the risk of child psychiatric disorders was significantly increased, although less so than in offspring whose parent had lung cancer. The most plausible explanation for our finding of an increased risk for child psychiatric disorder in offspring to parents with lung cancer seems to be parental smoking or other associated environmental exposures. Smoking is known to be associated with lower social status and educational level in Denmark, which might influence the associations. However, the finding that parental lung cancer was associated with an increased risk of child psychiatric disorders was still significant after adjusting for a parental history of psychiatric illness, which could be used as a proxy for social factors. The findings could also reflect the fact that parents with lung cancer had a higher risk of dying than parents with other types of cancers [53]. Furthermore, the temporal association with paternal and not maternal cancer could be due to the apparently increased mortality associated with paternal cancer, which has been suggested by other studies [53,54].

### Strengths and limitations

The study took advantage of the population-based nationwide registers in Denmark. One of the strengths of these registers are that all exposures were recorded independently of the outcome, and therefore not subject to recall bias. The classification and registration of schizophrenia in the Psychiatric Central Register was assumed to be comparable throughout the study period, although the ICD-8 was used to classify diagnoses until 1994, and the ICD-10 from 1994 onwards. During the two different periods, no changes have been reported in basic epidemiological characteristics, such as sex, age, family history and urbanization of persons with schizophrenia diagnosed in Denmark [55,56]. In order to make the child psychiatric diagnoses comparable throughout the study period, we only included diagnoses of a psychiatric disorder made before the age of 15. 

The unknown period between pregnancy and the initiation of maternal inflammatory reactions as a response to the cancer is a limitation of the study. The lack of statistical power when investigating the temporality is a result of the rarity of small-cell lung cancer and other cancers in mothers whose child later developed a psychiatric disorder during our study period. Furthermore, it is a limitation of our study that we do not have enough statistical power to look at specific child psychiatric diagnoses as autism and OCD, which are suspected to be of neurodevelopmental origin. One might also consider the psychological consequences for a child whose parent is suffering from a serious cancer diagnosis, and whether it would increase the risk of child psychiatric disorders. Since the diagnosis of cancer was mainly made many years after the childbirth, the cancer disease would probably not have influenced the upbringing with regard to the overall associations. However, since there was no overall temporal associations with parental cancer diagnoses, it does not seem that psychological stress during upbringing after a severe life threatening diagnosis as cancer in the parent affects the risk of developing schizophrenia or child psychiatric disorders. 

## Conclusion and Perspectives

Our findings do not in general support the hypothesis that cancer in the mother, and the initial development of the cancer with a possible immune response, might affect the child during critical periods of brain development in pregnancy. However, maternal small-cell lung cancer did show associations in subgroups but we could only study the associations in relatively few cases and whether the small-cell lung cancer was actually producing autoantibodies at the time of pregnancy remains unclear and speculative, but still hypothetically interesting in relation to other research showing associations between maternal immune responses during pregnancy and the risk of schizophrenia [2,4,47–49]. 

The risk of child psychiatric disorders was similarly increased in offspring of both mothers and fathers with lung cancer, and is therefore not likely to be induced by the immune system, but could be due to other environmental exposures of the fetus such as smoking, although there was little association with other smoking related cancers. Furthermore, our findings of no general association between parental cancers with schizophrenia or child psychiatric disorders do not indicate a genetic association. Maternal immune responses together with autoantibodies might be involved in the development of neurodevelopmental psychiatric disorders; however, this cannot be evaluated further in the present data. Studies with direct measurements of intrauterine exposure to specific autoantibodies and other immune components would be preferable. Cancer, even in its earliest presymptomatic stage, is very rare in mothers during pregnancy; and non-paraneoplastic autoantibodies are more likely to be involved in the possible association with neurodevelopmental psychiatric disorders.
